# Probable secondary hemophagocytic lymphohistiocytosis manifesting as central nervous system lesions after COVID-19 vaccination: a case report

**DOI:** 10.3389/fneur.2024.1363072

**Published:** 2024-03-11

**Authors:** Ju Hye Kim, Ji Yeon Chung, Jeong Bin Bong

**Affiliations:** Department of Neurology, Chosun University College of Medicine, Gwangju, Republic of Korea

**Keywords:** hemophagocytic lymphohistiocytosis, COVID-19 vaccines, ChAdOx1 nCoV-19, natural killer cell, acute disseminated encephalomyelitis

## Abstract

**Background:**

Hemophagocytic lymphohistiocytosis (HLH) is a rare systemic inflammatory disease commonly characterized by histiocyte infiltration in multiple organs, such as the liver, spleen, lymph nodes, bone marrow, and central nervous system. The clinical features of HLH include fever, splenomegaly, cytopenia, hypertriglyceridemia, hypofibrinogenemia, and elevated blood ferritin levels. HLH is categorized as either primary or secondary. Coronavirus disease 2019 (COVID-19) vaccines may occasionally trigger secondary HLH, which is related to hyperinflammatory syndrome.

**Case presentation:**

A 58-year-old woman, previously diagnosed with Graves’ disease, presented with cognitive decline 2 weeks after receiving the first dose of the ChAdOx1 nCoV-19 vaccine. Brain MRI revealed a hyperintense lesion on T2-weighted and fluid-attenuated inversion recovery images in the bilateral subcortical white matter and right periventricular area. Vaccination-associated acute disseminated encephalomyelitis was suspected and methylprednisolone and intravenous immunoglobulin (IVIg) were administered. From the 5th day of IVIg administration, the patient developed fever and pancytopenia. In the findings of bone marrow biopsy, hemophagocytosis was not observed; however, six of the eight diagnostic criteria for HLH-2004 were met, raising the possibility of HLH. Although there was no definitive method to confirm causality, considering the temporal sequence, suspicion arose regarding vaccine-induced HLH. Splenectomy was considered for therapeutic and diagnostic purposes; however, the patient died on the 28th day of hospitalization owing to multiple organ failure.

**Conclusion:**

To date, 23 cases of COVID-19 vaccine-related HLH have been reported. Additionally, HLH in COVID-19 patients has been reported in various case reports. To the best of our knowledge, this is the first reported case of central nervous system involvement in HLH related to any type of COVID-19 vaccine. This case suggests that even when there are no systemic symptoms after COVID-19 vaccination, HLH should be considered as a differential diagnosis if brain lesions are suggestive of CNS demyelinating disease.

## 1 Introduction

Hemophagocytic lymphohistiocytosis (HLH) is a rare immune disorder resulting from histiocyte infiltration into multiple organs, such as the liver, spleen, lymph nodes, bone marrow, and central nervous system (CNS). HLH is characterized by fever, cytopenia, splenomegaly, hypertriglyceridemia, hypofibrinogenemia, and elevated blood ferritin levels (1). HLH can be classified as primary or secondary. CNS involvement is less common among patients with secondary HLH compared with those with primary HLH. HLH can be difficult to differentiate from other disorders because infiltration of the CNS may manifest as an array of nonspecific neurological symptoms, including cranial nerve palsy, elevated intracranial pressure, and an altered level of consciousness (2). Brain magnetic resonance imaging (MRI) findings may also vary (2). As a result, delayed diagnosis of HLH with neurological manifestations is common, resulting in a poor prognosis. In this case report, we described a woman with secondary HLH with neurological manifestations following Coronavirus disease 2019 (COVID-19) vaccination with the Oxford Astra-Zeneca^®^ ChAdOx1 nCoV-19 vaccine.

## 2 Case presentation

**Figure 1:**
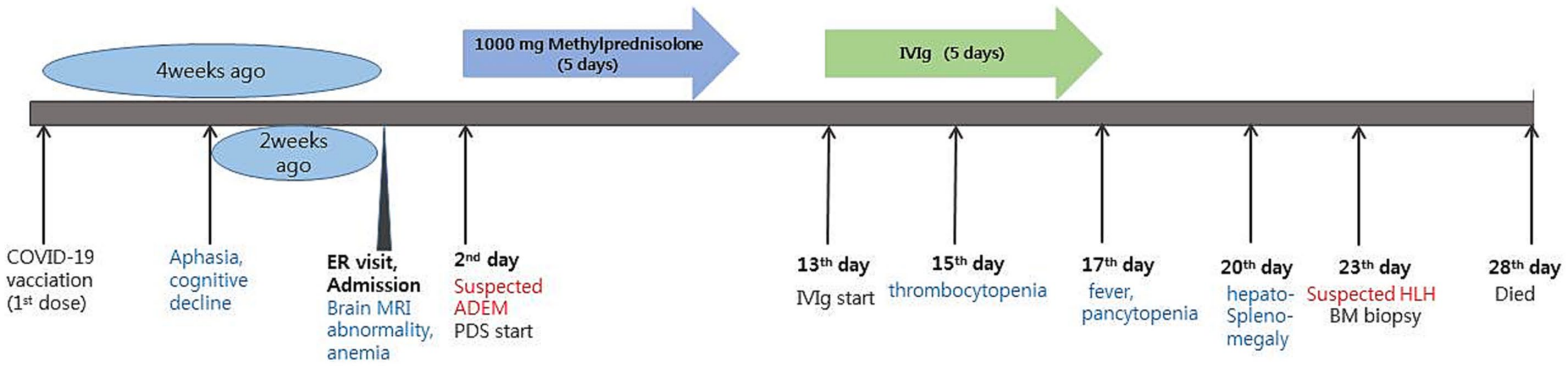
Timeline of the patient’s disease and treatment course. ER, emergency room; ADEM, acute disseminated encephalomyelitis; PDS, prednisolone; IVIg, intravenous immunoglobulin; HLH, hemophagocytic lymphohistiocytosis.

A 58-year-old female patient presented to the hospital with the chief complaints of aphasia and cognitive decline that began 2 weeks prior to presentation (Figure 1). Four weeks prior to the hospital visit, the patient had received the first dose of the COVID-19 vaccine (ChAdOx1 nCoV-19, Oxford Astra-Zeneca^®^). The patient did not experience any adverse events during the vaccination. In terms of medical history, she had been diagnosed with Graves’ disease 20 years prior and had received medical treatment at that time. Subsequently, the patient achieved complete remission and has not been taking any medications since then.

**Figure 2:**
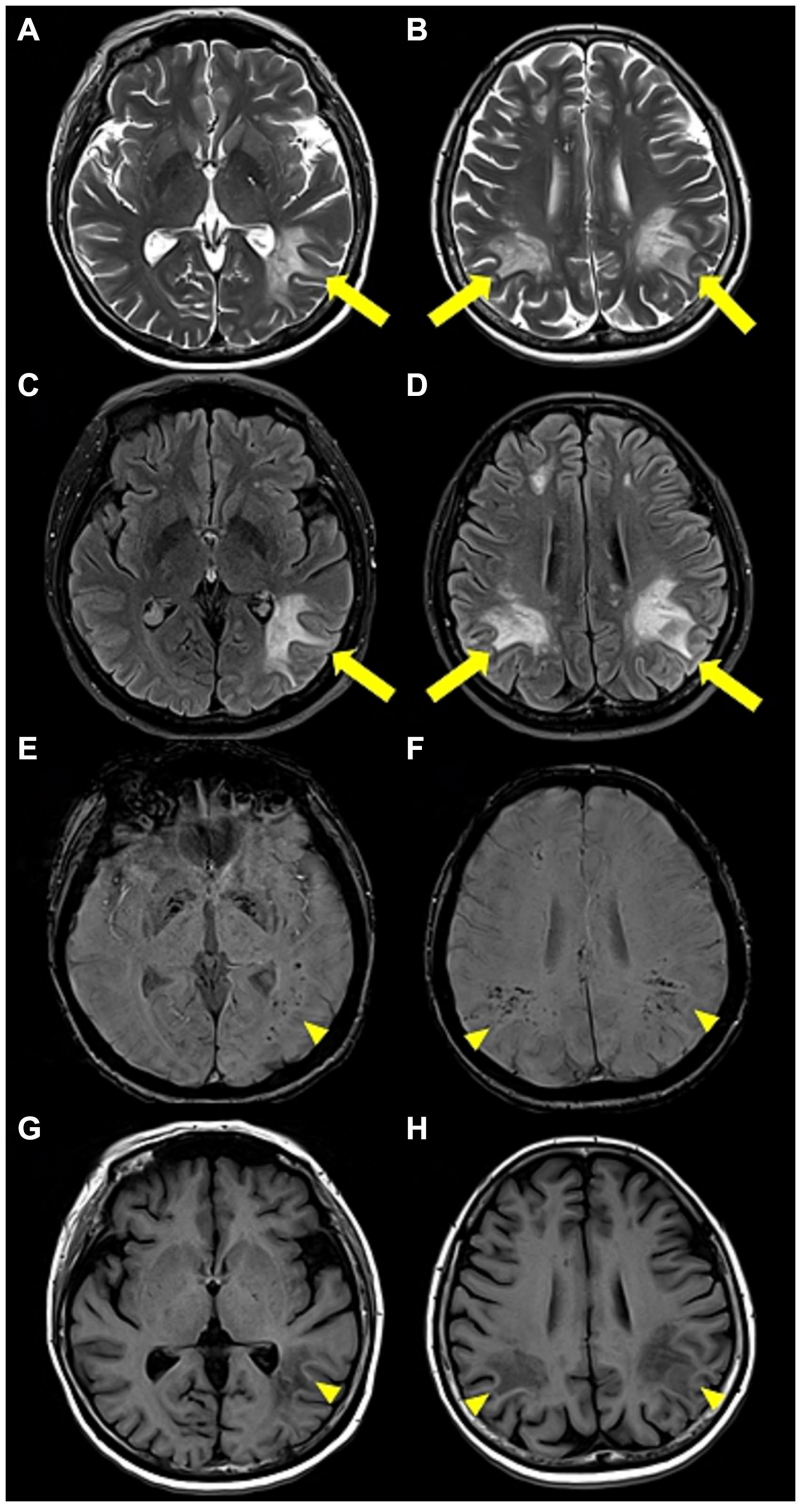
Brain magnetic resonance images. **(A,B)** Axial T2-weighted and **(C,D)** axial fluid-attenuated inversion recovery images revealed high-signal intensity lesions (yellow arrow) in the bilateral subcortical white matter and the right periventricular area. **(E,F)** Axial susceptibility-weighted images and **(G,H)** axial T1-weighted images showed low-signal intensity lesions (yellow arrowhead) in the same area.

Upon admission, the patient showed stable vital signs and was alert. Neurological examination revealed no limb weakness, sensory deficits, or cranial nerve deficits. Language assessment revealed transcortical aphasia and agraphia, with dyscalculia and finger agnosia. Blood test results were unremarkable, except for a decrease in hemoglobin level (Hb; 9.4 g/dL; normal range, 12–16 g/dL). Brain MRI revealed hyperintensity in the bilateral subcortical white matter and right periventricular area on both T2-weighted and fluid-attenuated inversion recovery images. Susceptibility-weighted imaging (SWI) revealed hypointensity in the same lesion area, and diffusion restriction was also observed on diffusion-weighted imaging, although without contrast enhancement (Figure 2). Analysis of a cerebrospinal fluid (CSF) sample showed a white blood cell count of 4/mm^3^, protein of 43.6 mg/dL, and glucose level of 66 mg/dL (serum glucose, 154 mg/dL). The finding of a CSF to serum glucose ratio of 0.5 or less was not considered a significant finding, as it is likely attributed to a time delay in the lumbar puncture procedure. Considering the current test results, post-vaccination acute disseminated encephalomyelitis (ADEM) was suspected, and 1 g of methylprednisolone was administered intravenously daily for 5 days. Spinal cord MRI was performed to differentiate it from other demyelinating disorders of the CNS; however, the findings were unremarkable. The IgG index was 0.49, and CSF oligoclonal bands, serum anti-aquaporin 4 antibodies, and serum anti-myelin oligodendrocyte glycoprotein antibodies were negative. Following high-dose steroids therapy, the patient scored 21 on the Korean mini-mental state examination, showing a 3-point improvement in the “attention and calculation” domain compared to before steroid therapy. Aphasia also improved, and the patient was able to speak words and simple sentences. Moreover, the patient was able to perform one-digit addition and subtraction. However, cognitive decline persisted. Hence, intravenous immunoglobulin (IVIg) therapy was initiated at 400 mg/kg/day on day 13, lasting for 5 days. Agraphia and cognitive function improved slightly during IVIg therapy, but her platelet level dropped to 10.8 K on day 3 of IVIg administration (admission day 15), and a fever of ≥38°C persisted from day 5 of IVIg administration (admission day 17). Blood tests indicated pancytopenia (white blood cell count, 3,920/mm^3^; Hb, 6.8 g/dL; and platelet count, 44,000/mm^3^). The C-reactive protein level was 8.5 mg/dL (normal range 0.0–0.3 mg/dL), and procalcitonin was 0.54 ng/mL (normal range 0–0.5 ng/mL). Blood, sputum, and urine cultures were performed to identify the cause of fever, but findings were unremarkable. The serum viral antibody test results were negative. CSF samples were tested using polymerase chain reaction and were negative for herpes simplex virus, varicella-zoster virus, and Epstein–Barr virus. Chest and abdominal computed tomography did not indicate infection, but showed hepatomegaly and splenomegaly.

Subsequently, the patient was referred to the hemato-oncology department for fever of unknown cause, and pancytopenia that lasted more than a week. HLH was suspected. Additional blood tests and bone marrow biopsies were also performed. Bone marrow biopsy did not indicate hemophagocytosis. However, six out of the eight HLH-2004 diagnostic criteria were met including fever, splenomegaly, cytopenia (Hb 6.8 g/dL and platelets 44,000/mm^3^), hypertriglyceridemia (triglycerides 268 mg/dL), increased ferritin (>1,675 mcg/L), and reduced natural killer cell (NK cell) activity (<40.0 pg./mL). As a results, the patient was diagnosed with probable HLH following COVID-19 vaccination, based on the temporal sequence, although it was not definitively proven. Splenectomy was planned to assess the preexisting causes of HLH (other than vaccination) and treatment. Unfortunately, the patient died on day 28 owing to sudden hypotension and multiple organ failure.

## 3 Discussion

Our patient presented with acute aphasia and cognitive decline 2 weeks after receiving the first dose of the COVID-19 vaccine. Subsequent brain MRI findings suggested ADEM, which led to the administration of high-dose steroids and IVIg. However, during the course of treatment, the patient developed systemic symptoms including fever and pancytopenia, eventually resulting in a diagnosis of HLH.

HLH is classified as either primary HLH (pHLH) or secondary HLH (sHLH) (3). pHLH predominantly affects children and is caused by genetic mutations that influence immune inactivation signaling pathways. In contrast, sHLH is triggered by infections, autoimmune disorders, and malignant tumors. Both pHLH and sHLH can involve the CNS, although CNS involvement is more common in pHLH (4). Neurological involvement may manifest as an array of nonspecific symptoms, including cranial nerve palsy, elevated intracranial pressure, and an altered level of consciousness. Moreover, brain MRI findings are not specific, so it is often difficult to differentiate HLH from CNS demyelinating disorders, such as ADEM or vasculitis of the CNS (2). Diagnosis of HLH may be delayed if only CNS symptoms manifest initially, or systemic symptoms develop a considerable time following the initial CNS symptom onset (1). CNS involvement indicates poor prognosis (5). In our case, the patient initially experienced CNS symptoms without systemic symptoms following a recent COVID-19 vaccination, leading to a provisional diagnosis of ADEM. However, approximately 4 weeks after the initial CNS symptoms, systemic symptoms presented, raising the suspicion of HLH. With a delay in diagnosis, multi-organ failure occurs, resulting in death.

A previous study was conducted to classify the differences in MRI findings among patients with pediatric pHLH, pediatric sHLH, and ADEM (4). In this study, comparison of the brain MRI scans of patients with pHLH and sHLH revealed that hypointense but clear lesions were observed on T1-weighted images more frequently in sHLH than in pHLH. Other distinct patterns emerged in the comparative analyses of the pHLH, sHLH, and ADEM groups. Compared to pHLH, ADEM cases exhibited more prominent lesions in the periventricle, brainstem, hypothalamus, and basal ganglia, displaying a higher frequency of hypointense signal intensity on T1-weighted images and an asymmetric distribution. In contrast, patients with pHLH manifested larger lesions than those with ADEM. The juxtacortical lesions were more distinctly visible in the ADEM group than in the sHLH group (4). Furthermore, a previous report showed that hemorrhagic brain lesions can sometimes be observed in HLH, possibly due to ischemic injury and necrosis from perivascular infiltration in systemic inflammation (6). In our patient, hypointense lesions on T1-weighted imaging and hypointense hemorrhagic lesions on SWI strongly suggested sHLH, rather than pHLH or ADEM. In cases showing lesions suspicious for CNS demyelinating and hemorrhagic lesions, even in the absence of typical systemic symptoms, HLH should be considered as a potential differential diagnosis.

To date, several cases of HLH following COVID-19 vaccination have been reported. mRNA-based COVID vaccines such as BNT162b2mRNA (Pfizer-BioNTech^®^) or mRNA-1273 (Moderna Therapeutics) have shown potential to trigger hyper-inflammatory syndrome due to an excessive secretion of interleukin-1-beta by dendritic cells in response to the spike protein encoded by the mRNA vaccine (7). Furthermore, adenovirus vector-based COVID vaccines, such as ChAdOx1 nCov-19 (AstraZeneca-Oxford) and Ad24.COV2.S (Janssen-Johnson & Johnson), may also potentially induce hyper-inflammatory syndrome, either as a reaction to the COVID-19 spike protein similar to mRNA vaccines or due to an exaggerated host immune response to the adenoviral component of the vaccine (8). To date, 23 cases of COVID-19 vaccine-related HLH have been reported (7–21) (Table 1). Of these, six patients developed HLH following ChAdOx1 nCov-19 vaccination, similar to our patient. Moreover, all six patients, including ours, developed HLH after the first dose of the vaccine. Thirteen of 23 patients developed HLH following BNT162b2mRNA vaccination (nine after the first dose and four after the second dose). Two patients developed HLH following the first dose of mRNA-1273 vaccination, one after Ad24.COV2.S vaccination, and the other after the first dose of inactivated severe acute respiratory syndrome coronavirus 2 vaccine. Among the 23 patients, twenty initially presented with fever, while one presented with appetite loss, lethargy, and rash without fever. In contrast, one patient with pre-existing well-controlled human immunodeficiency virus infection showed hypothermia (30.2°C) at the time of presentation (14). Another patient had an altered level of consciousness and dysarthria without fever, and was diagnosed with a transient ischemic attack. However, this patient developed fever 1 month later, and was eventually diagnosed with HLH (16). Similarly, our case showed no fever upon admission, but fever occurred during hospitalization for treatment of brain lesions. However, one important difference from previous reports was the presence of brain lesions, indicating CNS involvement in HLH, as observed in our patient.

**Table 1:** Clinical characteristics and treatment outcomes of patients with HLH after COVID-19 vaccination.

Case (ref.)	Age/sex	Vaccine	1st/2nd	Symptoms onset after vaccination	Underlying disease	HLH-2004 diagnostic criteria^a^ (number of criteria to be satisfied)	Hscore	Treatment	Outcomes
Case 1 (10)	68/M	ChAdOx1nCov-19	1st	10 days	Hypertension Gout Bowen’s disease	1,2,3,4,6	250	No therapy	Spontaneous improvement
Case 2 (12)	36/F	ChAdOx1nCov-19	1st	9 days	None	1,2,4	200	Methylprednisolone, IVIg	Improvement within 72 h, 2nd episode after 6 days, improved after IVIg
Case 3 (21)	71/F	ChAdOx1nCov-19	1st	7 days	Hypertension	1,2,3,4,5,6,8	293	Dexamethasone Etoposide	Discharged after 8 weeks in good condition
Case 4 (20)	60s/M	ChAdOx1nCov-19	1st	5 days	Hypertension	1,4,5,6,8,	259	Methylprednisolone IVIg Anakinra	Alive
Case 5 (20)	70s/F	ChAdOx1nCov-19	1st	7 days	Essential thrombocythemia Breast cancer in remission	1,4,5,6,8	220	Methylprednisolone IVIg Anakinra	Death
Case 6 (20)	30s/M	ChAdOx1nCov-19	1st	8 days	Ankylosing spondylitis	1,2,4,5,6,8	219	Methylprednisolone	Alive
Case 7 (21)	20/M	BNT162b2 mRNA	1st	2 days	None	1,2,3,4,5,6,7,8	229	Dexamethasone	Alive
Case 8 (13)	85/M	BNT162b2 mRNA	1st	Shortly	None	5,6	N/A	N/A	N/A
Case 9 (14)	52/M	BNT162b2 mRNA	1st	1 day	T-cell lymphoma	1,2,4,5,6,8	239	Dexamethasone Etoposide	Death (neutropenic fever and Bacteroides bacteremia)
Case 10 (14)	53/M	BNT162b2 mRNA	1st	4 days	Interstitial lung disease	1,4,6,8	213	Dexamethasone IVIg Anakinra	Alive
Case 11 (14)	55/F	BNT162b2 mRNA	1st	3 days	MAC, MDS pulmonary aspergillosis	1,2,3,4,8	208	Anakinra	Slowly recovered
Case 12 (7)	24/F	BNT162b2 mRNA	1st	10 days	None	1,2,3,5	259	Dexamethasone IVIg Anakinra	Discharged 14 days after treatment initiation in good condition
Case 13 (16)	60/M	BNT162b2 mRNA	1st	6 days	Barrett’s esophagus	1,2,3,4,6,8	198	Prednisone Etoposide	Partial remission
Case 14 (18)	14/F	BNT162b2 mRNA	1st	15 days	None	1,2,3,4,5,6	N/A	IVIg Methylprednisolone	Alive
Case 15 (17)	85/F	BNT162b2 mRNA	1st	12 days	Hypertension, Nephrosclerosis	1,3,4,6	N/A	Methylprednisolone	Alive
Case 16 (15)	38/F	BNT162b2 mRNA	2nd	21 days	None	1,4,6,7,8	147	Methylprednisolone	Discharged after 1 week after recovered within weeks
Case 17 (9)	21/M	BNT162b2 mRNA	2nd	14 days	None	1,2,3,4,5,6	319	Methylprednisolone Dexamethasone	Discharged 23 days after treatment initiation with good condition
Case 18 (16)	32/F	BNT162b2 mRNA	2nd	52 days	None	1,2,3,4,5,6,7	271	Dexamethasone Etoposide	Slowly recovered
Case 19 (19)	33/M	BNT162b2 mRNA	2nd	3 days	Hyperlipidemia allergies	1,2,3,4,6	274	Prednisone Methylprednisolone Anakinra IVIg	Death (acute liver failure)
Case 20 (14)	48/F	mRNA-1273	1st	8 days	HIV disseminated MAC and IRIS	1	130	Prednisone Infliximab	Improvement within 72 h
Case 21 (14)	57/M	mRNA-1273	1st	12 days	Controlled HIV	3,4,8	185	Methylprednisolone	Death
Case 22 (11)	43/F	Chinese inactivated SARS-CoV-2 vaccine	1st	Shortly	EBV	1,3,4,5,6,7	261	Dexamethasone	Discharged 17 days after start of dexamethasone
Case 23 (8)	61/unknown	Ad26.COV2.S	1st	10 days	Hypertension, Warthin’s right parotid gland tumor	1,2,3,4,5,6	186	Dexamethasone IVIg	Death (acute respiratory insufficiency, cardiorespiratory arrest- 46 days after vaccination)

IVIg, intravenous immunoglobulin; MAC, Mycobacterium avium complex; MDS, myelodysplastic syndrome; HIV, human immunodeficiency virus; IRIS, immune reconstitution inflammatory syndrome; EBV, Epstein-Varr virus; mRNA, messenger RNA; N/A, not applicable.

a HLH 2004 diagnostic critereia: 1—Fever (≥ 38.3°C); 2—splenomegaly; 3—cytopenia in ≥ 2 lines (Hb < 9 g/dL, plt < 100/nL, neutrophils < 1.0/nL); 4—ferritin ≥ 500 μg/L; 5—hypertriglyceridemia and/or hypofibrinogenemia (fasting triglycerides ≥ 265 mg/dL, fibrinogen < 1.5 g/L); 6—hemophagocytosis in bone marrow or spleen or lymph nodes; 7—low or absent Natural killer cell activity; 8—soluble CD25 (soluble IL-2 receptor) ≥ 2,400 U/mL.

Treatment for HLH primarily begins with immunosuppressive agents like cyclosporine and dexamethasone to manage immune system hyperactivity. If this approach proves insufficient, cytotoxic chemotherapy agents are administered to target excessive T cells and NK cells. Options include etoposide-based regimens or cyclophosphamide, doxorubicin, vincristine, prednisolone (CHOP) therapy, commonly used in lymphoma. In cases of CNS involvement, intrathecal methotrexate injections may be considered. However, if patients do not respond to initial therapy, experience reactivation, or are diagnosed with primary HLH, allogeneic hematopoietic stem cell transplantation (HSCT) becomes necessary (22). The long-term survival rate after allogeneic HSCT is approximately 22–59% (23). In our case, initially misdiagnosed as ADEM, immunotherapies such as high-dose steroids and IVIg were administered, resembling initial immunotherapy for HLH; however, these treatments proved ineffective. Therefore, prompt diagnosis of HLH is crucial for timely initiation of aggressive treatment, including cytotoxic agents. Unfortunately, our study of the present case had several limitations. Firstly, we were unable to perform a splenectomy for therapeutic and diagnostic purposes to confirm hemophagocytosis in the spleen. Additionally, we could not perform an autopsy or brain histopathology examination to exclude other causes and definitively confirm sHLH with CNS involvement. To the best of our knowledge, this is the first report of sHLH with CNS involvement following vaccination with all types of COVID-19 vaccines, including the ChAdOx1 nCov-19.

In conclusion, sHLH should be considered as a differential diagnosis in cases that present with neurological symptoms without systemic symptoms, such as fever, following COVID-19 vaccination. This is important because these neurological symptoms could indicate CNS involvement related to HLH.

## Data availability statement

The original contributions presented in the study are included in the article/supplementary material, further inquiries can be directed to the corresponding author.

## Ethics statement

The study involving humans was approved by Chosun University Hospital IRB/2023-10-007. The study was conducted in accordance with the local legislation and institutional requirements. Written informed consent for participation was not required from the participants or the participants’ legal guardians/next of kin in accordance with the national legislation and institutional requirements. Written informed consent was obtained from the patient’s legal guardian for the publication of this case report.

## Author contributions

JK: Writing – original draft. JC: Writing – review & editing. JB: Writing – original draft, Writing – review & editing.
